# Ubiquitinylation of α-Synuclein by Carboxyl Terminus Hsp70-Interacting Protein (CHIP) Is Regulated by Bcl-2-Associated Athanogene 5 (BAG5)

**DOI:** 10.1371/journal.pone.0014695

**Published:** 2011-02-16

**Authors:** Lorraine V. Kalia, Suneil K. Kalia, Hien Chau, Andres M. Lozano, Bradley T. Hyman, Pamela J. McLean

**Affiliations:** 1 Department of Neurology, MassGeneral Institute for Neurodegenerative Disease, Massachusetts General Hospital, Harvard Medical School, Charlestown, Massachusetts, United States of America; 2 Division of Neurology, Toronto Western Research Institute, University Health Network, University of Toronto, Toronto, Canada; 3 Division of Neurosurgery, Toronto Western Research Institute, University Health Network, University of Toronto, Toronto, Canada; 4 Division of Brain, Imaging and Behaviour-Systems Neuroscience, Toronto Western Research Institute, University Health Network, University of Toronto, Toronto, Canada; University of Pittsburgh, United States of America

## Abstract

Parkinson's disease (PD) is a common neurodegenerative condition in which abnormalities in protein homeostasis, or proteostasis, may lead to accumulation of the protein α-synuclein (α-syn). Mutations within or multiplications of the gene encoding α-syn are known to cause genetic forms of PD and polymorphisms in the gene are recently established risk factors for idiopathic PD. α-syn is a major component of Lewy bodies, the intracellular proteinaceous inclusions which are pathological hallmarks of most forms of PD. Recent evidence demonstrates that α-syn can self associate into soluble oligomeric species and implicates these α-syn oligomers in cell death. We have previously shown that carboxyl terminus of Hsp70-interacting protein (CHIP), a co-chaperone molecule with E3 ubiquitin ligase activity, may reduce the levels of toxic α-syn oligomers. Here we demonstrate that α-syn is ubiquitinylated by CHIP both *in vitro* and in cells. We find that the products from ubiquitinylation by CHIP include both monoubiquitinylated and polyubiquitinylated forms of α-syn. We also demonstrate that CHIP and α-syn exist within a protein complex with the co-chaperone bcl-2-associated athanogene 5 (BAG5) in brain. The interaction of CHIP with BAG5 is mediated by Hsp70 which binds to the tetratricopeptide repeat domain of CHIP and the BAG domains of BAG5. The Hsp70-mediated association of BAG5 with CHIP results in inhibition of CHIP E3 ubiquitin ligase activity and subsequently reduces α-syn ubiquitinylation. Furthermore, we use a luciferase-based protein-fragment complementation assay of α-syn oligomerization to investigate regulation of α-syn oligomers by CHIP in living cells. We demonstrate that BAG5 mitigates the ability of CHIP to reduce α-syn oligomerization and that non-ubiquitinylated α-syn has an increased propensity for oligomerization. Thus, our results identify CHIP as an E3 ubiquitin ligase of α-syn and suggest a novel function for BAG5 as a modulator of CHIP E3 ubiquitin ligase activity with implications for CHIP-mediated regulation of α-syn oligomerization.

## Introduction

Parkinson's disease (PD) is a movement disorder affecting approximately three percent of the population over the age of sixty-five and is second only to Alzheimer's disease as the most common neurodegenerative disease [1]. Loss of dopaminergic neurons in the substantia nigra pars compacta is one of the neuropathological hallmarks of all forms of PD. In addition, idiopathic PD and most familial forms of PD are characterized by the presence of intracellular protein aggregates, known as Lewy bodies and Lewy neurites, within the surviving nigral neurons. α-Synuclein (α-syn) is a major component of these protein inclusions [2], [3]. Genetic evidence supports a role for α-syn in the pathogenesis of PD. In particular, missense mutations (A53T, A30P, and E46K) in the α-syn gene (PARK1), as well as duplications and triplications of the locus containing the α-syn gene (initially PARK4), are associated with rare familial forms of PD [4]. Furthermore, polymorphisms in the gene have recently been identified as risk factors for idiopathic PD [5], [6]. α-syn as well as other proteins within Lewy bodies are frequently ubiquitinylated [2], [3]. These inclusions also contain members of the heat shock protein (Hsp) family such as Hsp70 [7]–[9], and co-chaperone molecules including carboxyl terminus of Hsp70-interacting protein (CHIP) [10] and bcl-2-associated athanogene 5 (BAG5) [11].

Although α-syn-containing protein aggregates are a neuropathological feature of PD, there is considerable debate regarding the role of protein aggregates in neurodegenerative disorders including PD. Recent evidence suggests that α-syn not only forms insoluble deposits within Lewy bodies but that α-syn monomers can also self associate into soluble higher-order structures such as oligomers. These soluble oligomeric species of α-syn may confer significant toxicity to cells [12]–[17] which may be modulated by chaperones and co-chaperones [14], [18]–[20]. We have previously demonstrated that the co-chaperone CHIP associates with α-syn and reduces the levels of toxic α-syn oligomers via both lysosomal and proteasomal pathways [10], [14].

CHIP contains an amino terminal tetratricopeptide repeat (TPR) domain which mediates its interaction with both Hsp70 and Hsp90 [21], [22] and a carboxyl terminal U-box domain which confers E3 ubiquitin ligase activity [23], [24]. A number of substrates of CHIP-mediated E3 ubiquitin ligase have been identified including Hsp70 [25], glucocorticoid receptor [22], ErbB2 [26], neuronal nitric-oxide synthase (nNOS) [27], the mutant androgen receptor associated with spinal and bulbar muscular atrophy [28], and more recently leucine-rich repeat kinase-2 (LRRK2) [29], [30]. Members of the BAG domain-containing family of proteins have been shown to interact with CHIP and regulate its function [23], [31]–[33]. There are currently six known human BAG family members (BAG1 to BAG6) which functionally interact with diverse binding partners and regulate important processes such as cell division and cell death. We have previously shown that BAG5 may enhance dopaminergic neuronal death in models of PD [11]. Here we identify BAG5 as a negative regulator of CHIP. We find that α-syn is a novel substrate of CHIP and demonstrate that BAG5 both inhibits α-syn ubiquitinylation by CHIP and mitigates CHIP-mediated reduction of α-syn oligomers.

## Results

### CHIP Reduces α-Synuclein Oligomerization

**Figure 1:**
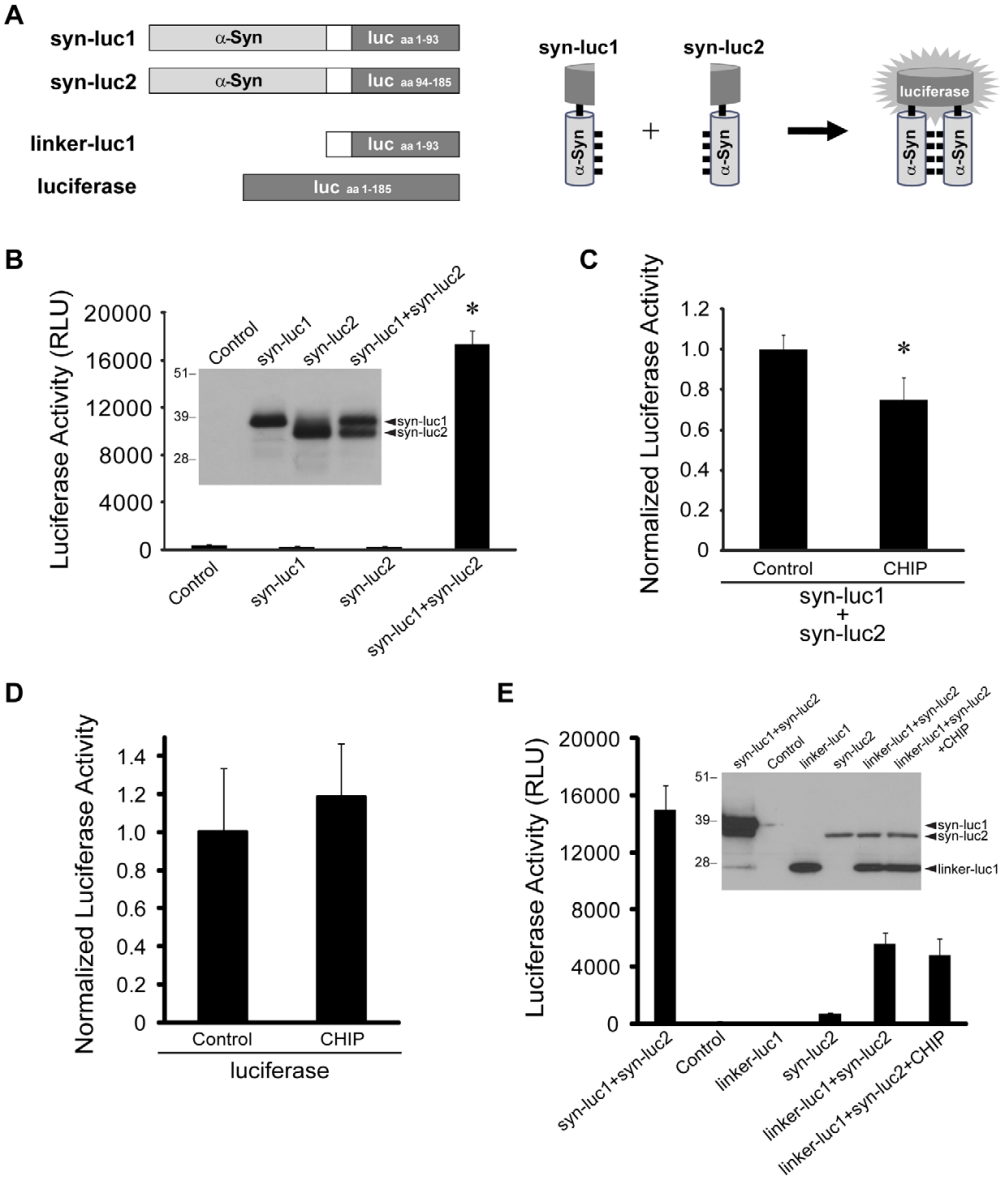
CHIP overexpression decreases α-syn oligomer levels. (A) Schematic of the constructs used in the bioluminescent PCA with *Gaussia princeps* luciferase (luc) to assay for α-syn oligomerization. Fragments of luc were cloned downstream of full-length human α-syn to generate the expression constructs referred to as syn-luc1 and syn-luc2. Reconstitution of luc from the fragments can occur upon α-syn-α-syn interactions in cells and thus luciferase activity provides a surrogate measure of α-syn oligomerization [16], [19]. Control experiments were performed with a luc fragment without α-syn (linker-luc1) and with full-length luciferase. (B) Luciferase activity was measured from H4 cells transfected with pcDNA control, syn-luc1 or syn-luc2 alone, or co-transfected with syn-luc1 and syn-luc2. Bars correspond to mean (± S.D.) luciferase activity measured in relative luciferase units (RLU). *P<0.05, ANOVA with Tukey Honest Significance Difference (HSD) post hoc test versus pcDNA control. Results are representative of three experiments performed in triplicate. Protein expression of syn-luc1 and syn-luc2 was analyzed by Western blot with anti-α-syn antibodies (inset). Molecular weight markers are shown in kDa. (C) Luciferase activity was measured from H4 cells co-transfected with syn-luc1 and syn-luc2 plus either pcDNA control or CHIP. Bars correspond to mean (± S.D.) luciferase activity normalized to measures obtained for co-transfection of syn-luc1 and syn-luc2 with pcDNA. *P<0.01, t-test versus pcDNA control. Results are representative of three experiments performed in triplicate. (D) Luciferase activity was measured from cells co-transfected with full-length luciferase plus either pcDNA control or CHIP. There was no statistically significant difference between these two conditions (P>0.05, t-test versus pcDNA control). (E) PCAs were performed with the indicated constructs. There was no statistically significant difference between luciferase activity in cells co-expressing linker-luc1 and syn-luc2 with or without CHIP (P>0.05, t-test). Protein expression was examined by Western blot with anti-luc antibodies (inset).

We have recently developed a highly sensitive bioluminescent protein-fragment complementation assay (PCA) to study the regulation of α-syn oligomerization [16], [19], [34]. In this strategy, full-length human α-syn was subcloned upstream from the optimized amino terminal (amino acids 1–93) or carboxyl terminal (amino acids 94–185) fragments of *Gaussia princeps* luciferase to generate the expression constructs referred to as syn-luc1 and syn-luc2, respectively (Fig. 1A). Reconstitution of a complete luciferase molecule from the fragments can occur upon α-syn-α-syn interactions in cells and thus luciferase activity provides a surrogate measure of α-syn oligomerization [16], [19]. In human H4 neuroglioma cells, transient transfection of syn-luc1 or syn-luc2 individually did not demonstrate any luciferase activity over transfection with the control vector pcDNA whereas co-expression of syn-luc1 and syn-luc2 showed a significant increase in measurable luciferase activity relative to baseline (Fig. 1B). We found that co-expression of CHIP with syn-luc1 and syn-luc2 resulted in a 25% reduction in luciferase activity compared with control vector (Fig. 1C). Thus, we inferred that CHIP decreases the levels of α-syn oligomers.

Nevertheless, it is possible that CHIP with its co-chaperone function might reduce luciferase activity by directly altering the folding or stability of luciferase independent of α-syn. This possibility was tested in experiments using constructs of the amino terminal fragment without α-syn, referred to as linker-luc1, or of the full-length *Gaussia princeps* luciferase (Fig. 1A). Co-expression of CHIP with full-length luciferase did not result in a reduction in luciferase activity compared with control vector (Fig. 1D). Furthermore, we found that the low level of protein complementation that occurred with co-expression of linker-luc1 and syn-luc2 was not significantly altered by overexpression of CHIP (Fig. 1E). The results from these experiments demonstrated that CHIP does not modulate the luciferase activity of full-length luciferase or of reconstituted luciferase in the absence of α-syn-α-syn interactions, indicating that the reduction of luciferase activity in the PCAs is due to the effect of CHIP on α-syn oligomers.

### CHIP Mediates Ubiquitinylation of α-Synuclein

**Figure 2:**
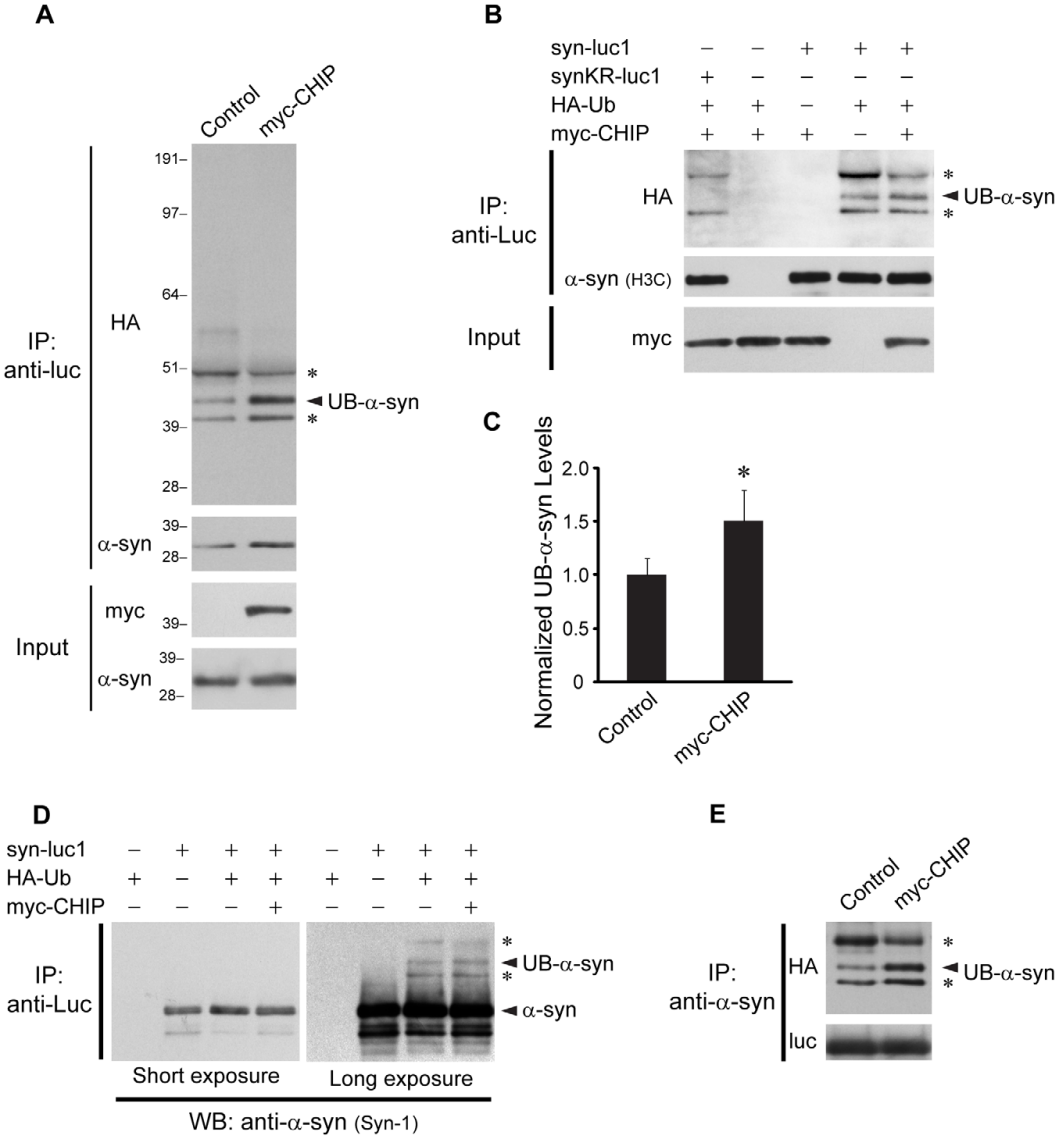
CHIP mediates ubiquitinylation of α-syn in cells. (A) Immunoprecipitations with anti-luc were performed from lysates of H4 cells transfected with HA-Ub, syn-luc1, and control vector or myc-CHIP as indicated. Immunoprecipitates were sequentially probed with anti-HA (upper) and anti-α-syn (middle) antibodies. Five percent of lysates used for immunoprecipitation was loaded as input and probed with anti-myc or anti-α-syn antibodies (lower). The middle band represents monoubiquitinylated α-syn (UB-α-syn). The asterisks (*) indicate immunoprecipitated bands that remain detectable by the anti-HA antibody following the substitution of all lysines within the α-syn sequence (see (B)). Molecular weight markers are indicated on left in kDa. (B) Immunoprecipitations with anti-luc were performed from lysates of H4 cells transfected with syn-luc1, synKR-luc1, HA-Ub, and myc-CHIP as indicated. Immunoprecipitated proteins were probed with anti-α-syn antibodies (H3C) which recognizes both syn-luc1 and synKR-luc1. The asterisks (*) correspond with the same bands indicated as such in (A). (C) Densitometric quantification of the band representing monoubiquitinylated α-syn when co-transfected with a control vector or myc-CHIP was performed from three independent experiments, one of which is represented in (A). Bars correspond to mean (± S.D.) gray value normalized to measures obtained for co-transfection of syn-luc1 and HA-Ub with control vector. *P<0.05, t-test versus control vector. (D) Proteins immunoprecipitated with anti-luc were probed with anti-α-syn antibodies (Syn-1) with a short or long exposure to film. The asterisks (*) correspond with the same bands seen in (A) and (B). (E) Immunoprecipitations with anti-α-syn were performed from lysates of H4 cells transfected with syn-luc1 and HA-Ub with control vector or myc-CHIP. Immunoprecipitated proteins were sequentially probed with anti-HA (upper) and anti-luc (lower) antibodies. The asterisks (*) correspond to the bands as indicated in (A), (B), and (D). Results are representative of three experiments.

Given that CHIP regulates α-syn oligomerization and is also known to associate in a complex with α-syn [14], we hypothesized that α-syn may be a substrate of CHIP-mediated E3 ubiquitin ligase activity. To explore this hypothesis, we first performed a series of immunoprecipitation experiments from lysates of H4 cells co-transfected with HA-tagged ubiquitin (HA-Ub) and syn-luc1 or syn-luc2. The tags of either syn-luc1 (Fig. 2A) or syn-luc2 (data not shown) allowed for immunoprecipitation from transfected cell lysates with an anti-luciferase (anti-luc) antibody. We performed immunoprecipitation under strongly denaturing conditions to prevent the possible co-immunoprecipitation of ubiquitinylated proteins other than α-syn. Using this approach, we found three major bands on Western blot which were immunoreactive with anti-HA antibodies (Fig. 2A). To identify which, if any, of these immunoprecipitated bands corresponded to ubiquitinylated forms of α-syn rather than the luciferase fragment, we designed a mutant of human full-length α-syn which was resistant to ubiquitinylation by mutating all potential canonical ubiquitinylation sites in α-syn. To generate this mutant α-syn, we substituted the lysine residues within α-syn with arginine, which is similar in size and charge to lysine. This mutant gene was then subcloned upstream from the amino terminal or carboxyl terminal fragments of luciferase to generate synKR-luc1 and synKR-luc2, respectively. These constructs were identical to syn-luc1 and syn-luc2 with the exception of the lysine-to-arginine substitutions within α-syn only. The mutant synKR-luc1, like wild-type syn-luc1, could be immunoprecipitated by anti-luc antibodies and was detected on Western blot with an anti-α-syn antibody (H3C) which binds to an epitope that contains no lysines within the carboxyl terminal region of α-syn. We discovered that one of the three bands from syn-luc1 immunoprecipitates detected by anti-HA antibodies was not present in synKR-luc1 immunoprecipitates, indicating that this band corresponds to a ubiquitinylated form of α-syn in cells (Fig. 2B). In contrast, the other two bands represent either ubiquitinylation of lysine residues within the luciferase fragment tag only or amino terminal ubiquitinylation of α-syn [35]. Given that α-syn is not a likely candidate for amino terminal ubiquitinylation because it is acetylated at the amino terminus [36], [37], the latter is a less likely possibility.

Using densitometric analysis of relative grey values in multiple experiments (n = 3), we found that ubiquitinylation of α-syn was increased by approximately 50% with co-expression of CHIP (Fig. 2C). Similar results were found when the immunoprecipitates were probed with anti-α-syn antibodies at longer exposure (Fig. 2D) or when α-syn was immunoprecipitated by anti-α-syn instead of anti-luc antibodies (Fig. 2E).

### CHIP E3 Activity is Sufficient for Ubiquitinylation of α-Synuclein

**Figure 3:**
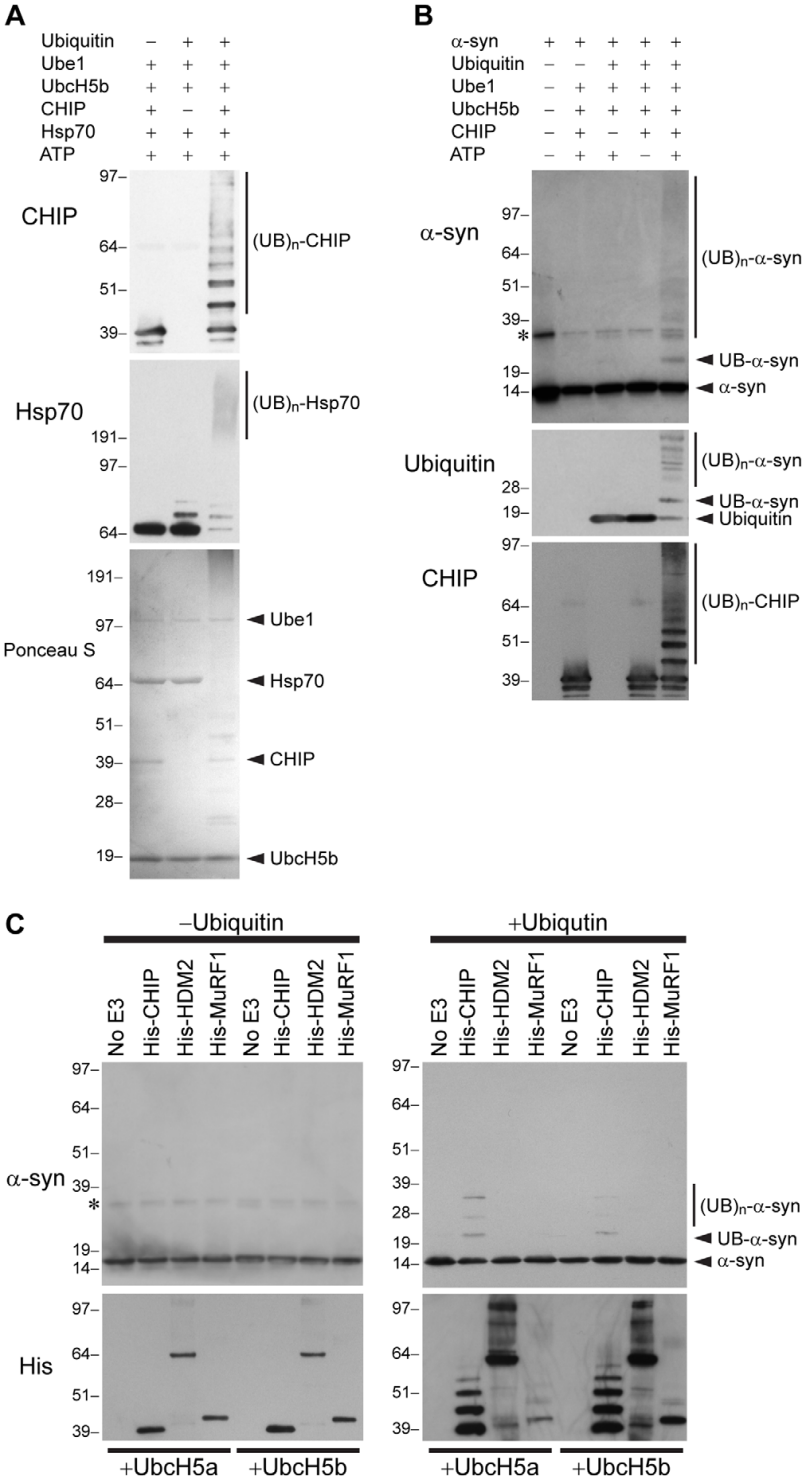
Wild-type α-syn is a substrate of CHIP E3 ubiquitin ligase activity *in vitro*. (A) *In vitro* ubiquitinylation assays were performed by incubating purified recombinant proteins of the E1 Ube1, the E2 UbcH5b, and the CHIP substrate Hsp70 with and without purified ubiquitin or CHIP. Ubiquitinylation of CHIP and Hsp70 was determined by Western blot using anti-CHIP (upper) and anti-Hsp70 (middle) antibodies, respectively. The high molecular weight smears represented ubiquitinylated ((UB)_n_) forms of CHIP and Hsp70. The purified recombinant proteins used in the assay were stained with Ponceau S (lower). Similar results were found in more than five experiments. Molecular weight markers are shown on left in kDa. (B) *In vitro* ubiquitinylation assays were performed by incubating purified recombinant human untagged wild-type α-syn with ubiquitin, Ube1, UbcH5b, CHIP, and Mg-ATP as indicated. Ubiquitinylation of α-syn (UB-α-syn and (UB)_n_-α-syn) was determined by Western blot using anti-α-syn (upper) and anti-ubiquitin (middle) antibodies. The asterisk (*) indicates a non-specific band. Ubiquitinylation of CHIP was detected with anti-CHIP antibodies (lower). Results are representative of more than five experiments. Molecular weight markers are shown in kDa. (C) *In vitro* ubiquitinylation assays were performed with α-syn, Ube1, and either the E2 UbcH5a or UbcH5b plus His-tagged CHIP, HDM2, or MuRF1 or in the absence of an E3 ubiquitin ligase. Experiments were performed without and with ubiquitin as indicated. Ubiquitinylation of α-syn and the E3 ubiquitin ligases was detected with anti-α-syn and anti-His antibodies, respectively. The asterisk (*) indicates a non-specific band. Molecular weight markers in kDa are indicated.

A given substrate may be ubiquitinylated by any one or more of the multiple E3 ubiquitin ligases expressed within cells. In addition to its E3 activity, CHIP has also been shown to have E4-like activity in that it positively regulates other E3s such as parkin [38]. To test whether CHIP E3 activity is sufficient for ubiquitinylation of α-syn, we utilized a reconstituted *in vitro* system in which we combined purified recombinant CHIP, in the absence of any other E3s, with purified ubiquitin, the E1 Ube1, and the E2 UbcH5b. Like many E3 ubiquitin ligases, CHIP is known to ubiquitinylate itself *in vitro* [24]. We first confirmed CHIP E3 ligase activity in our system by testing for auto-ubiquitinylation of CHIP, as well as ubiquitinylation of Hsp70, a known substrate of CHIP [25] (Fig. 3A). Given that the overexpression of CHIP enhanced ubiquitinylation of syn-luc1 in cells, we next determined whether untagged wild-type α-syn was a substrate of CHIP by testing purified wild-type α-syn in this *in vitro* system. We detected by Western blot a molecular weight band of approximately 24 kDa in size recognized by both anti-α-syn and anti-ubiquitin antibodies (Fig. 3B). Higher molecular weight ubiquitinylated α-syn species were also recognized by these antibodies. CHIP E3 activity was verified in these assays by CHIP auto-ubiquitinylation (Fig. 3B).

To test the specificity of α-syn ubiquitinylation by CHIP in our system, we examined two other E3 ubiquitin ligases, HDM2 [39] and MuRF1 [40], which utilize the same UbcH5 family of E2s as CHIP to mediate the ubiquitinylation of substrate proteins. Using HDM2 or MuRF1 with the E2s UbcH5b or UbcH5a, each E3 was enzymatically active as demonstrated by their ability to auto-ubiquitinylate. However, we found that neither HDM2 nor MuRF1 mediated the ubiquitinylation of α-syn *in vitro* (Fig. 3C). Taken together, these results imply that untagged wild-type α-syn is a substrate of CHIP and that CHIP E3 ubiquitin ligase activity is sufficient for α-syn ubiquitinylation.

### α-Synuclein is Monoubiquitinylated by CHIP

**Figure 4:**
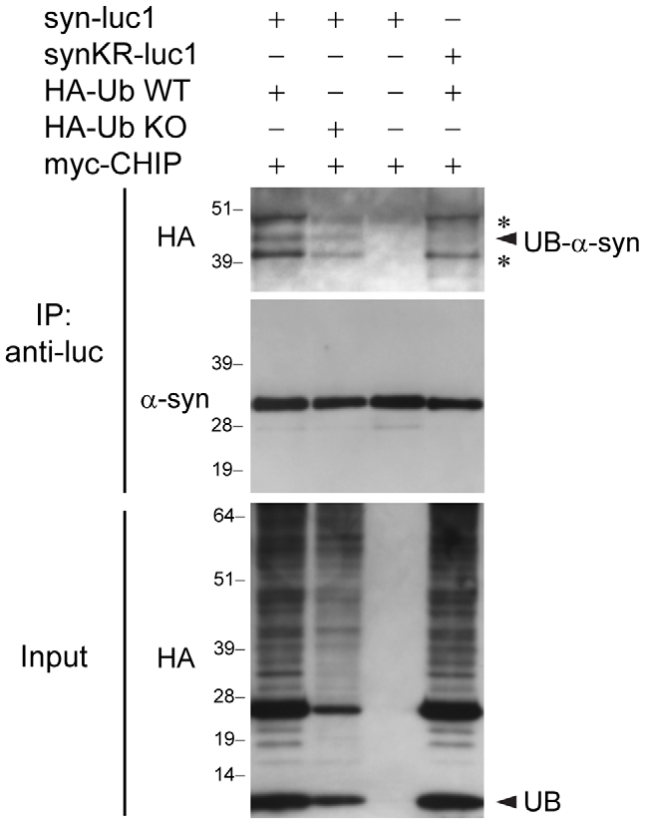
CHIP monoubiquitinylates α-syn. Immunoprecipitations with anti-luc were performed from lysates of H4 cells transfected with syn-luc1, synKR-luc1, HA-Ub WT, HA-Ub KO, and myc-CHIP as indicated. Immunoprecipitates were sequentially probed with anti-HA (upper) and anti-α-syn (Syn-1) (middle) antibodies. For the anti-α-syn blots, short exposure times were used to allow for the comparison of amount of α-syn immunoprecipitated in each of the conditions. Ubiquitinylated forms of α-syn are not detectable by Syn-1 at these shorter exposure times (see Figure 2D). Five percent of lysates used for immunoprecipitation was loaded as input and probed with anti-HA antibodies which recognize HA-ubiquitin monomers (UB) and proteins with covalently attached HA-ubiquitin (lower). The middle band represents monoubiquitinylated α-syn (UB-α-syn). The asterisks (*) correspond with immunoprecipitated bands that remain detectable by the anti-HA antibody following the substitution of all lysines within the α-syn sequence. These are the same bands seen in Figure 2. Similar results were found in each of three experiments.

One of the ubiquitinylated forms of α-syn identified in the *in vitro* ubiquitinylation assays with CHIP was approximately 24 kDa in size (Fig. 3B and 3C) which is compatible with α-syn linked to monoubiquitin. To investigate the possibility that α-syn may be monoubiquitinylated in cells, we utilized a mutant of ubiquitin that lacks lysine residues (HA-UbKO) thus preventing polyubiquitinylation, but not monoubiquitinylation, of substrates. We found that with co-transfection of HA-Ub KO, in place of wild-type ubiquitin, the same band we identified as corresponding to ubiquitinylated α-syn was present (Fig. 4). Thus, CHIP may both polyubiquitinylate and monoubiquitinylate α-syn.

### CHIP Interacts with the Co-Chaperone BAG5

The identity of molecules responsible for regulating CHIP-mediated ubiquitinylation of α-syn and reduction of α-syn oligomerization by CHIP is unknown. A molecular candidate for modulating CHIP function is the BAG domain-containing co-chaperone BAG5. The activity of CHIP has been shown to be regulated by members of the BAG family, including BAG1 [23] and BAG2 [32], [33], [41] which each associate with CHIP and modulate CHIP E3 ubiquitin ligase function. We have previously discovered that BAG5, a BAG family member which is unique in that it contains multiple BAG domains, enhances α-syn-mediated toxicity in cultured cells and dopaminergic neuronal death in models of PD [11]. The E3 ubiquitin ligase parkin is negatively regulated through an interaction with BAG5 [11]. Therefore, we tested whether CHIP may also interact with BAG5.

**Figure 5:**
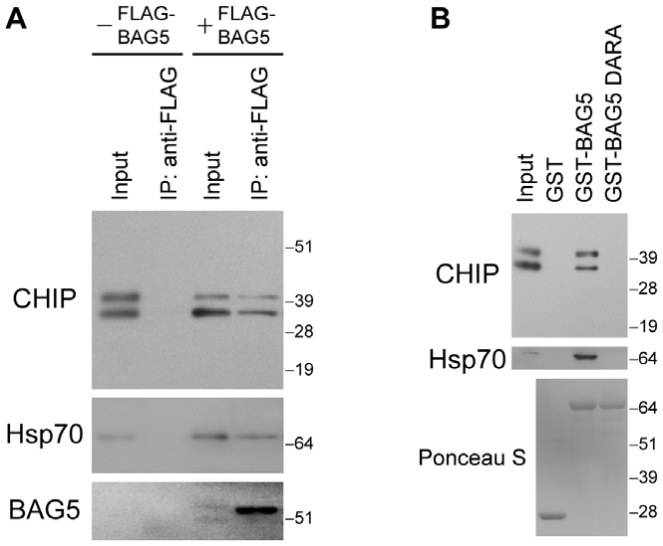
CHIP forms a protein complex with BAG5. (A) Immunoprecipitations with anti-FLAG antibodies were performed from lysates of H4 cells transfected with CHIP with or without FLAG-BAG5 as indicated. Immunoprecipitates were sequentially probed with anti-CHIP (upper), anti-Hsp70 (middle), and anti-FLAG (lower) antibodies. Ten percent of lysates used for immunoprecipitation was loaded as input. The upper CHIP band corresponds to monoubiquitinylated CHIP [62]. Molecular weight markers are shown on right. Similar results were found in three separate experiments. (B) PDAs were performed using lysates of H4 cells transfected with CHIP. Proteins that associated with GST alone, GST-BAG5, or GST-BAG5 DARA were probed with anti-CHIP (upper) and anti-Hsp70 (middle) antibodies. Input was 10% of lysates used for PDAs. The presence of equal amounts of GST fusion proteins was confirmed by Ponceau S staining of the membranes (lower). Molecular weight markers are indicated on right. Results are representative of four independent experiments.

We expressed CHIP with or without FLAG-tagged BAG5 (FLAG-BAG5) in H4 neuroglioma cells and found that CHIP co-immunoprecipitated with FLAG-BAG5 but not with anti-FLAG antibodies in the absence of FLAG-BAG5 (Fig. 5A). Given that we have previously shown that BAG5 interacts with Hsp70 [11], the same membranes were re-probed with anti-Hsp70 antibodies which confirmed the presence of endogenous Hsp70 in the immunoprecipitates with FLAG-BAG5 and its absence in the immunoprecipitates lacking FLAG-BAG5. We next performed pull down assays (PDAs) with GST fusion proteins using lysates from H4 cells transiently transfected with full-length CHIP. We demonstrated that a purified recombinant GST fusion protein of BAG5 (GST-BAG5) pulled down full-length CHIP (Fig. 5B). In contrast, CHIP was not pulled down by GST alone, confirming that the specificity of the interaction is conferred by BAG5 and not the GST tag. CHIP was also not pulled down by a GST fusion protein of a mutant of BAG5 called BAG5 DARA (see below).

### Hsp70 Mediates the Association between CHIP and BAG5

**Figure 6:**
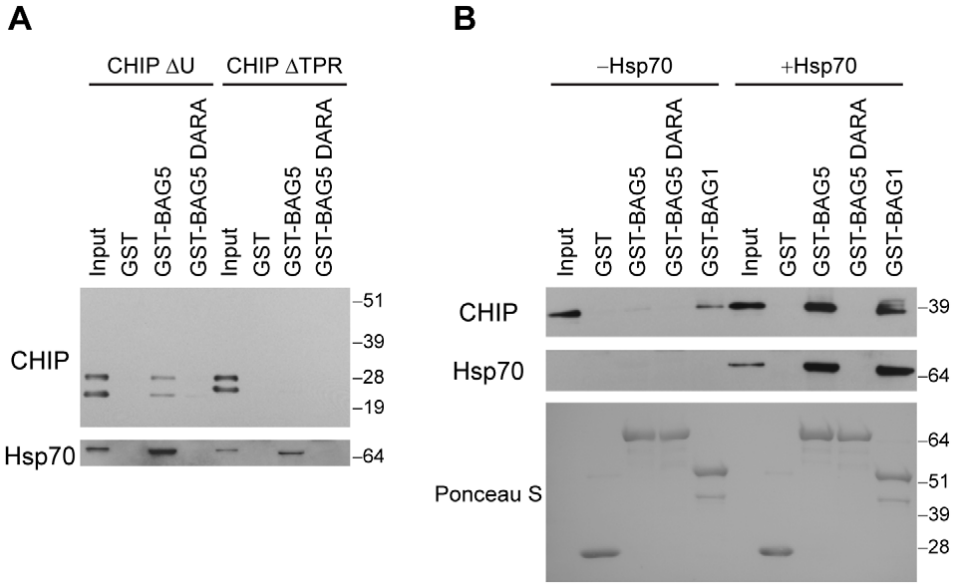
Hsp70 is required for the association between CHIP and BAG5. (A) PDAs with GST fusion proteins using lysates of H4 cells transfected with the CHIP deletion constructs CHIP ΔU and CHIP ΔTPR are shown. The blots were probed with anti-CHIP (upper) and anti-Hsp70 (lower) antibodies. The inputs shown are 10% of total lysates used in each assay. Molecular weight markers are indicated. Results are representative of three independent experiments. (B) PDAs were performed using GST fusion proteins and purified recombinant CHIP with or without Hsp70 as indicated. Proteins that associated with GST alone, GST-BAG5, GST-BAG5 DARA, or GST-BAG1 were probed with anti-CHIP (upper) and anti-Hsp70 (middle) antibodies. Input was 10% of proteins used for PDAs. The presence of equal amounts of GST fusion proteins was confirmed by Ponceau S staining (lower). Molecular weight markers are indicated. Results are representative of three independent experiments.

To map which domain of CHIP may mediate the interaction with BAG5, we performed PDAs using lysates from H4 cells individually transfected with CHIP deletion constructs lacking the U-box domain (CHIP ΔU) or the TPR domain (CHIP ΔTPR). We found that GST-BAG5 pulled down CHIP ΔU but not CHIP ΔTPR (Fig. 6A). PDAs with GST alone served to control for the GST tag of GST-BAG5 and demonstrated no interaction with either CHIP ΔU or CHIP ΔTPR. To ensure that the purified recombinant BAG5 protein was in its native conformation and hence would have interacted with BAG domain ligands, we probed the proteins pulled down by GST-BAG5 for endogenous Hsp70 (Fig. 6A), which is known to directly bind to the BAG domains of BAG5 [11], [42].

The TPR domain of CHIP is known to mediate an interaction with Hsp70 [43]. Given that its deletion prevented the association of CHIP with BAG5, we hypothesized that Hsp70 may mediate the interaction between CHIP and BAG5. We have previously characterized a mutant of BAG5 called BAG5 DARA which does not interact with Hsp70 but retains the ability to dimerize with itself, to heterodimerize with wild-type BAG5, and to interact with other BAG5 binding partners [11]. To explore the possibility that Hsp70 mediates the CHIP-BAG5 interaction, we used a GST fusion protein of BAG5 DARA (GST-BAG5 DARA) in PDAs. We found that GST-BAG5 DARA, which did not pull down endogenous Hsp70 (Fig. 5B), also did not pull down full-length CHIP (Fig. 5B) or the CHIP deletion mutants (Fig. 6A).

To further test our hypothesis, we performed PDAs in an isolated *in vitro* system using purified recombinant CHIP and Hsp70 proteins. We found that a GST fusion protein of BAG1 (GST-BAG1) pulled down CHIP in the absence of Hsp70 (Fig. 6B). BAG1 is a BAG domain-containing protein previously shown to directly interact with CHIP but to also have enhanced binding to CHIP in the presence of Hsp70 [23]. In contrast to GST-BAG1, we found that like GST alone, neither GST-BAG5 nor GST-BAG5 DARA pulled down significant amounts of CHIP in the absence of Hsp70. However, with the addition of Hsp70, GST-BAG5 pulled down CHIP whereas GST alone or GST-BAG5 DARA did not (Fig. 6B). From these results, we conclude that CHIP indirectly associates with BAG5. The association between CHIP and BAG5 is mediated by Hsp70 which is known to bind to the TPR domain of CHIP and the BAG domains of BAG5.

### BAG5 Inhibits the Ubiquitinylation of α-Synuclein by CHIP

**Figure 7:**
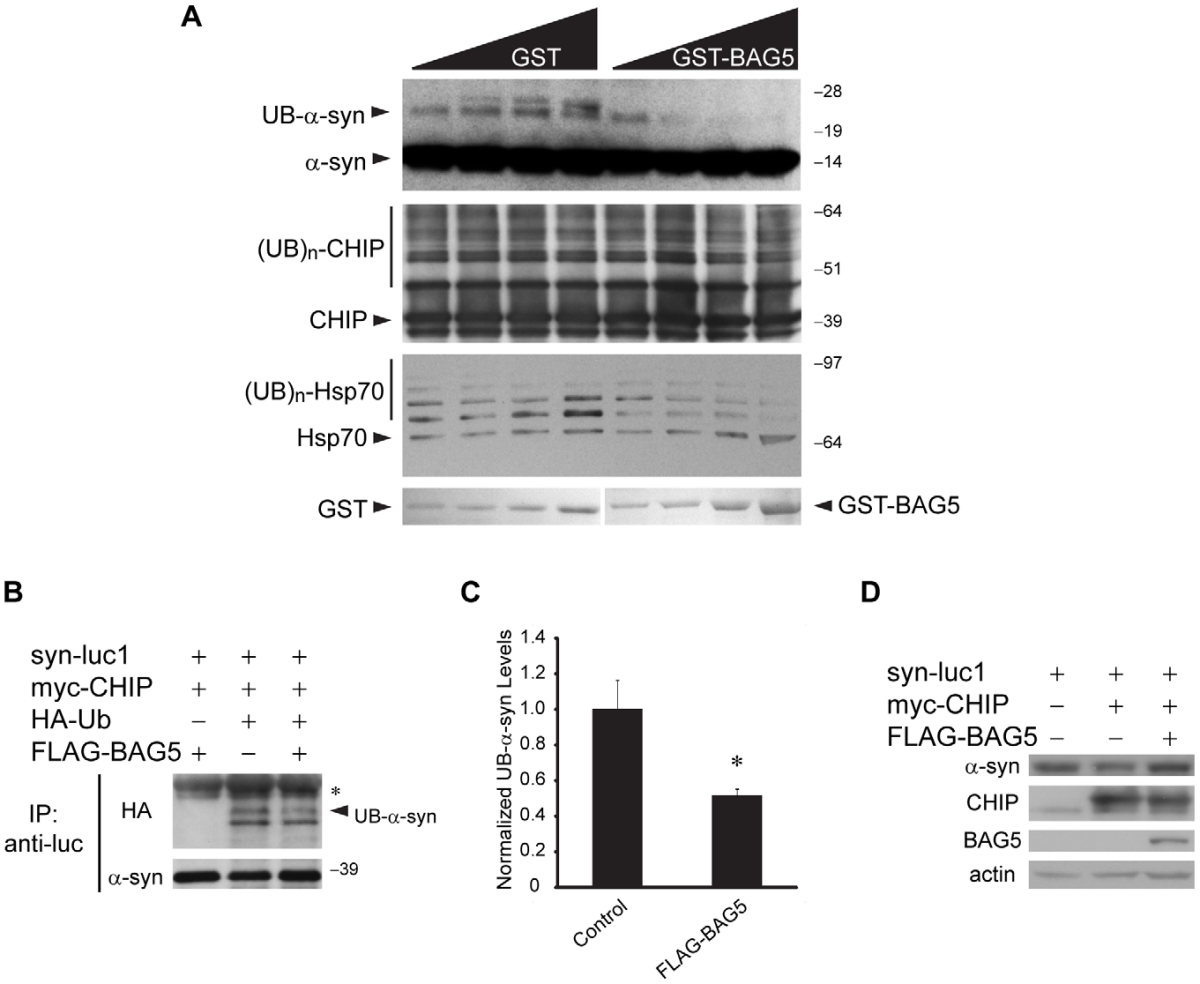
BAG5 inhibits CHIP-mediated ubiquitinylation of α-syn *in vitro* and in cells. (A) *In vitro* ubiquitinylation assays were performed with increasing amounts of GST or GST-BAG5 as indicated. Ubiquitinylation was determined by Western blot using anti-α-syn, anti-CHIP, or anti-Hsp70 antibodies. GST fusion proteins were stained with Ponceau S. Similar results were found in three experiments. (B) Immunoprecipitations with anti-luc were performed from lysates of H4 cells transfected with syn-luc1, myc-CHIP, HA-Ub, and FLAG-BAG5 as shown. Ubiquitinylation of α-syn was detected using anti-HA antibodies (upper). The arrow indicates the monoubiquitinylated form of α-syn (UB-α-syn). Immunoprecipitation of equivalent amounts of α-syn was confirmed by probing with anti-α-syn antibodies (lower). The asterisk (*) indicates cross-reactivity of the secondary antibody with the immunoglobulin light chain. (C) Quantification by densitometric analysis of the UB-α-syn band with co-transfection of a control vector or FLAG-BAG5 from three independent experiments, one of which is represented in (B). Bars shown correspond to mean (±S.D.) gray value normalized to measures obtained for the control vector. *P<0.05, t-test versus control vector. (D) Protein expression levels of α-syn and CHIP with and without BAG5 overexpression was assessed by Western blot. Actin was used as a loading control.

To begin to investigate the consequences of the CHIP-BAG5 association on CHIP function, we examined whether BAG5 affects the E3 ubiquitin ligase activity of CHIP. We found that addition of purified recombinant GST-BAG5 to *in vitro* ubiquitinylation assays with CHIP and α-syn resulted in a significant and dose-dependent reduction in α-syn monoubiquitinylation compared to the addition of equimolar amounts of GST (Fig. 7A). We also found a dose-dependent decrease in ubiquitinylation of Hsp70 with the addition of GST-BAG5. The levels of auto-ubiquitinylation of CHIP were unaltered regardless of the addition of GST or GST-BAG5 suggesting that the effect of BAG5 on CHIP-mediated ubiquitinylation may be specific for both α-syn and Hsp70. We next tested the effect of BAG5 on CHIP-mediated ubiquitinylation of α-syn in cells by performing immunoprecipitation experiments from lysates of H4 cells co-transfected with syn-luc1, myc-CHIP, HA-Ub, and FLAG-BAG5 (Fig. 7B). Co-expression of BAG5 with CHIP resulted in a significant decrease of approximately 50% in the ubiquitinylation levels of α-syn compared to empty control vector (Fig. 7C). Overexpression of BAG5 did not alter the levels of α-syn or CHIP (Fig. 7D). Together these findings indicate that BAG5 inhibits CHIP-mediated ubiquitinylation of α-syn.

### BAG5 Mitigates CHIP-Mediated Reduction of α-Synuclein Oligomers

**Figure 8:**
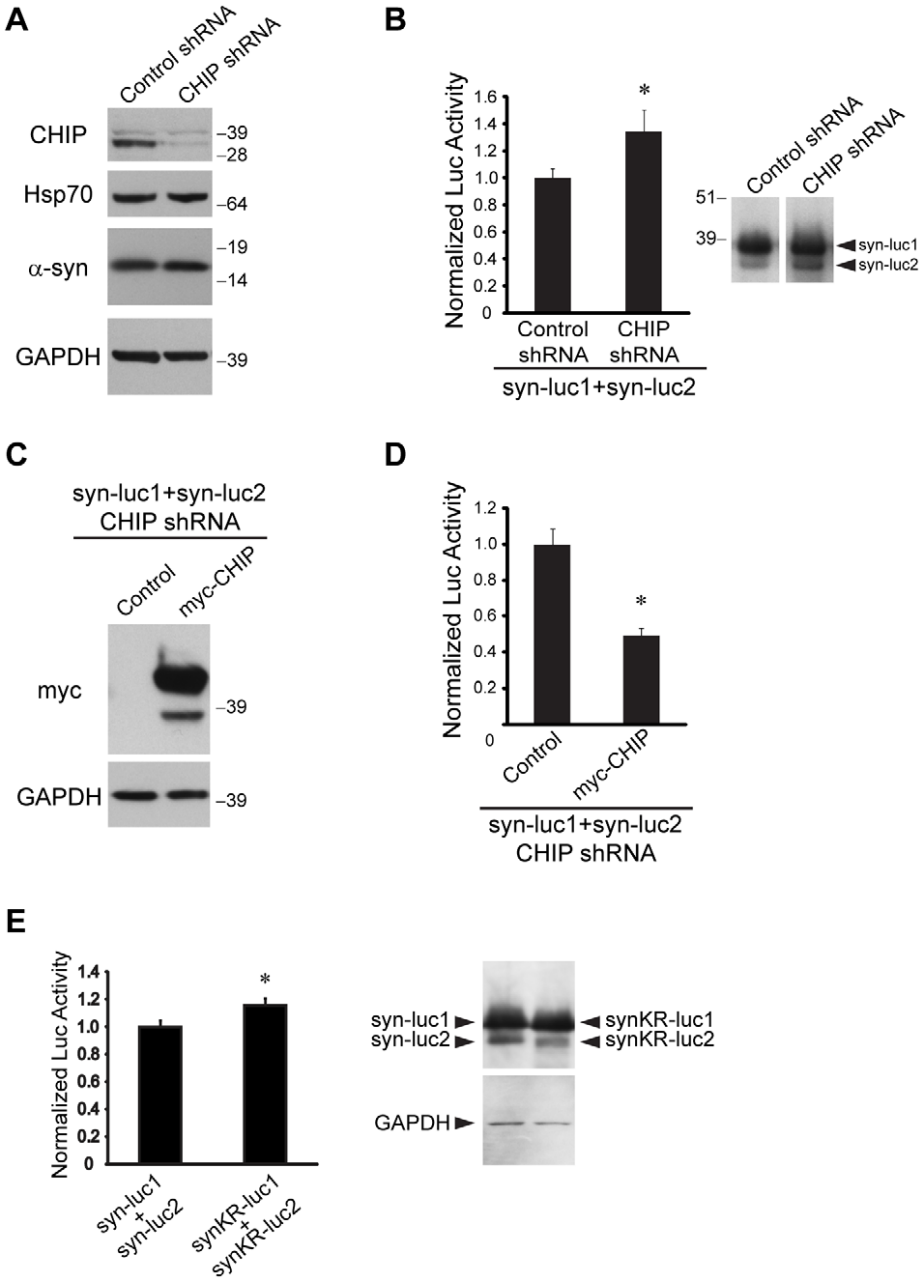
Targeted knockdown of CHIP or absence of α-syn ubiquitinylation increases oligomerization of α-syn. (A) Knockdown of CHIP protein expression in the CHIP shRNA stable cell line versus a control shRNA cell line was confirmed by Western blot. The membrane was sequentially probed with anti-CHIP, anti-Hsp70, and anti-α-syn antibodies. GAPDH was used as a loading control. (B) Luciferase activity was measured from CHIP shRNA or control shRNA stable cell lines transiently co-transfected with syn-luc1 and syn-luc2. Bars correspond to mean (± S.D.) luciferase activity normalized to measures obtained for control shRNA cells co-transfected with syn-luc1 and syn-luc2. *P<0.01, t-test versus control shRNA. Results are representative of three experiments performed in triplicate in three different stable cell line clones. Protein expression of syn-luc1 and syn-luc2 was analyzed by Western blot with anti-luc antibodies (inset). (C) CHIP shRNA stable cell line was transiently co-transfected with syn-luc1 and syn-luc2 plus either pcDNA as control or myc-CHIP. Protein expression of myc-CHIP was confirmed by Western blot with anti-myc antibodies and anti-CHIP antibodies (data not shown). GAPDH was used as a loading control. (D) Luciferase activity was measured from cells described in (C). Bars correspond to mean (± S.D.) luciferase activity normalized to measures obtained for co-transfection of syn-luc1 and syn-luc2 with pcDNA control. *P<0.01, t-test versus control. Results are representative of six experiments performed in triplicate. (E) Luciferase activity was measured from H4 cells transfected with syn-luc1 and syn-luc2 or with synKR-luc1 and synKR-luc2 relative to protein expression by densitometric quantitation. Bars correspond to mean (± S.D.) luciferase activity normalized to measures obtained for co-transfection with syn-luc1 and syn-luc2. *P<0.05, t-test versus syn-luc1+syn-luc2. Protein expression of syn-luc1, syn-luc2, synKR-luc1, and synKR-luc2 was analyzed by Western blot with anti-luc antibodies and GAPDH was used as a loading control (inset). Results are representative of three experiments performed in triplicate.

To explore the potential consequences of ubiquitinylation of α-syn by CHIP, we performed a series of experiments using the luciferase-based PCA to assess α-syn oligomerization. We first determined the effect of targeted reduction of CHIP activity on the levels of α-syn oligomers. To this end, we generated a stable H4 cell line in which CHIP protein expression was knocked down utilizing short-hairpin RNA (shRNA). We also made a control cell line which was stably transfected with a non-targeting shRNA vector that activates the RNA-induced silencing complex (RISC) and RNA interference (RNAi) pathway. We confirmed knockdown of CHIP protein expression by Western blot in the CHIP shRNA stable cell line relative to the control cell line (Fig. 8A). There were no differences in the expression of endogenous α-syn or Hsp70. We co-transfected the CHIP shRNA stable cell line or control cell line with syn-luc1 and syn-luc2. We found a significant increase of approximately 35% in measured luciferase activity in cells with a targeted reduction of CHIP expression relative to control cells (Fig. 8B). Similar results were obtained in three separate CHIP shRNA stable cell lines (data not shown). To confirm the specificity of the observed shRNA effect on CHIP, we utilized a myc-tagged CHIP expression vector which is resistant to the targeting shRNA (Fig. 8C). Co-transfecting syn-luc1 and syn-luc2 with myc-CHIP in the CHIP knockdown cell line resulted in a significant decrease in measured luciferase activity (Fig. 8D). Consistent with CHIP activity mediating reduction of α-syn oligomers, expression of myc-CHIP rescued the effect of CHIP shRNA knockdown and resulted in a larger decrease of α-syn oligomers in the CHIP knockdown cell line than in cells with baseline endogenous CHIP expression (see Fig. 1C).

Next we performed luciferase-based PCAs using the mutant α-syn constructs which have all lysine residues in α-syn substituted with arginine and consequently cannot be ubiquitinylated by CHIP. We found that co-expression of the mutants synKR-luc1 and synKR-luc2 resulted in a 16% increase in luciferase activity compared to co-expression of syn-luc1 and syn-luc2 (Fig. 8E), accounting for approximately 50% of the effect seen with the knockdown of CHIP activity (see Fig. 8B). Thus, knockdown of CHIP function increases the levels of oligomeric α-syn species and α-syn mutants that are not ubiquitinylated have a greater propensity to oligomerize.

**Figure 9:**
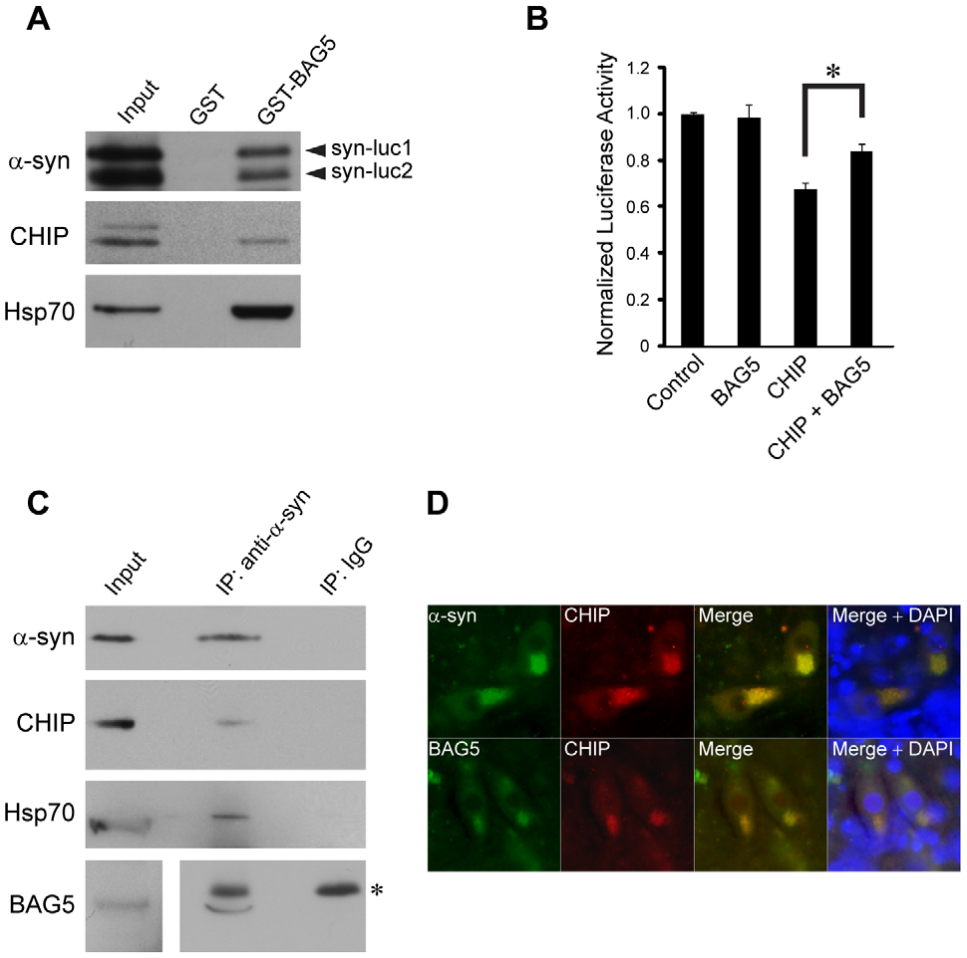
BAG5 negatively regulates CHIP-mediated reduction of α-syn oligomer levels. (A) PDAs were performed using GST fusion proteins and lysates from H4 cells transfected with syn-luc1, syn-luc2, CHIP, and Hsp70. Proteins that associated with GST alone or GST-BAG5 were probed with anti-α-syn (upper), anti-CHIP (middle), and anti-Hsp70 (lower) antibodies. Input was 10% of total lysates used for PDAs. Results are representative of three independent experiments. (B) Luciferase activity was measured from H4 cells transfected with syn-luc1 and syn-luc2 plus the vectors indicated on the horizontal axis of the graph. The total DNA used in each condition was equalized using the empty vector pcDNA. Bars correspond to mean (±s.e.m.) luciferase activity normalized to measures obtained for the co-transfection of syn-luc1 and syn-luc2 with pcDNA as control (*P<0.05, ANOVA with Tukey HSD post hoc test versus control). Results shown are from four independent experiments performed in triplicate. (C) Immunoprecipitations with anti-α-syn antibodies or IgG control were performed from mouse brain homogenates. Immunoprecipitates were sequentially probed with anti-α-syn, anti-CHIP, anti-Hsp70, and anti-BAG5 antibodies as indicated. Ten percent of lysates used for immunoprecipitation was loaded as input. A longer exposure of the same blot was required to visualize the input for BAG5. The asterisk (*) indicates cross-reactivity of the secondary antibody with the immunoglobulin heavy chain. Similar results were found in three separate experiments. (D) Immunohistochemistry for α-syn, BAG5, and CHIP was performed on substantia nigra pars compacta samples from patients with a neuropathological diagnosis of DLB. α-syn immunoreactivity (green in upper), BAG5 immunoreactivity (green in lower), and CHIP immunoreactivity (red) in neurons and in α-syn-positive intracytoplasmic inclusions are shown. Co-localization is demonstrated in the merged images (yellow). DAPI staining (blue) demonstrates nuclear localization. Five to ten percent of nigral neurons contained α-syn-positive intracytoplasmic inclusions. At least ten α-syn-positive inclusion-containing neurons were visualized in the substantia nigra pars compacta of each of two patients with DLB. All of the visualized α-syn-positive inclusions were positive for both BAG5 and CHIP.

Since BAG5 inhibits CHIP-mediated ubiquitinylation of α-syn, it is predicted that BAG5 would preclude the reduction of α-syn oligomers by CHIP. To test this prediction, we first performed PDAs to examine for protein-protein interactions and found that syn-luc1 and syn-luc2 may associate with the CHIP-Hsp70-BAG5 complex (Fig. 9A). Next, we found that in luciferase-based PCAs co-expression of BAG5 with CHIP blocked approximately 50% of the CHIP-mediated reduction of α-syn oligomers (Fig. 9B).

To examine whether an interaction may occur between endogenously expressed α-syn, CHIP, and BAG5, we performed co-immunoprecipitations using mouse brain homogenates and found that these proteins form a complex (Fig. 9C). Furthermore, we investigated whether these proteins may co-localize in neurons within human brain. We performed immunohistochemistry for α-syn, CHIP, and BAG5 on substantia nigra pars compacta samples from two patients with a neuropathological diagnosis of dementia with Lewy bodies (DLB), a neurodegenerative disease with histopathology similar to PD. CHIP immunoreactivity co-localized with both α-syn and BAG5 within α-syn-positive intracytoplasmic inclusions suggestive of Lewy bodies in the substantia nigra of both patients (Fig. 9D). Colocalization was also observed in the cytoplasm of neurons containing α-syn-positive inclusions as well as neurons lacking inclusions. Thus, we demonstrate an association of endogenous α-syn, CHIP, and BAG5. Taking our data together, we conclude that the CHIP-BAG5 interaction negatively regulates CHIP-mediated reduction of α-syn oligomerization.

## Discussion

There is growing evidence that a subpopulation of α-syn may be covalently modified by ubiquitinylation and that ubiquitinylated α-syn is present in Lewy bodies [44]–[46]. However, identification of the key enzymes involved and understanding of the functional consequences of α-syn ubiquitinylation remain less clear. Here we found that CHIP overexpression enhanced ubiquitinylation of α-syn in cells. To provide evidence that our finding was not a result of the action of other E3s or was not simply due to the previously demonstrated E4-like function of CHIP, we utilized an isolated *in vitro* system to test CHIP E3 activity. This approach had the added advantage of allowing us to study untagged wild type human α-syn. We found that CHIP, but not the other E3 ubiquitin ligases HDM2 or MuRF1, ubiquitinylates α-syn. Hence we show that α-syn is a substrate of the ubiquitin ligase CHIP and that CHIP E3 activity is sufficient to mediate α-syn ubiquitinylation. Future studies will be necessary to elucidate whether CHIP's putative E4 activity also plays a role in enhancing α-syn ubiquitinylation *in vivo*.

α-syn is a major component of Lewy bodies and these pathological protein inclusions are frequently immunoreactive for ubiquitin [3]. Protein extracts from the brains of patients with Lewy body pathology have been reported to contain monoubiquitinylated or multiubiquitinylated α-syn species in which a single ubiquitin moiety is attached to one or more lysine residues, respectively [36], [45], [46]. In the present study, we found that α-syn ubiquitinylated by CHIP *in vitro* migrated at a size of 24 kDa, which is consistent with the calculated size for a monoubiquitinylated form of α-syn, as well as at higher molecular weights. With polyubiquitinylation of a protein, ubiquitins are linked to each other by isopeptide bonds between lysine and carboxyl terminal glycine residues to form polyubiquitin chains. Monoubiquitinylation, but not polyubiquitinylation, can occur if all lysine residues are mutated in ubiquitin (Ub KO). We found that in cells, α-syn could be ubiquitinylated with wild-type ubiquitin or with mutant Ub KO by CHIP. Thus, the products from CHIP-mediated ubiquitinylation of α-syn include not only polyubiquitinylated but also monoubiquitinylated species.

Monoubiquitinylated α-syn was also identified in H4 cells without overexpression of CHIP. It is possible that this finding was partly a result of the appreciable endogenous expression of CHIP in these cells. Alternatively, functional redundancy of E3 ubiquitin ligases has been observed in *CHIP* ^−/−^ mouse embryonic fibroblasts in which established CHIP substrates, such as nNOS, are ubiquitinylated by other E3 ligases [47]. Similarly the E3 ligase seven in absentia homolog (SIAH) has previously been found to monoubiquitinylate α-syn [48], [49]. Knockdown of endogenous SIAH does not completely abolish α-syn ubiquitinylation [48] consistent with a possible contribution of several E3 ligases, including CHIP, to the monoubiquitinylation of α-syn.

Many substrates of E3 ubiquitin ligases have been identified as being monoubiquitinylated. However, the consequences of monoubiquitinylation for a substrate protein are continuing to be defined. Monoubiquitinylation has been implicated in the targeting of membrane proteins for endocytosis and lysosomal degradation [50] and in the regulation of protein complex assembly [51]. Monoubiquitinylation has also been found to act as a nuclear export signal [52] and to regulate transcription [50]. In contrast to polyubiquitinylation, monoubiquitinylation was initially thought to not participate in proteasomal degradation. However, monoubiquitinylation has been recently reported to act as a signal for targeting Pax3 to the proteasome [53]. The exact fate of monoubiquitinylated species of α-syn remains elusive. Whereas monoubiquitinylation of α-syn by SIAH promotes the formation of large insoluble intracellular aggregates [49], we have previously demonstrated that CHIP decreases insoluble α-syn aggregate formation [10]. Here we also found that CHIP reduces soluble α-syn oligomeric species. Thus there may be differential consequences of ubiquitinylation of α-syn depending on the specific E3 ubiquitin ligase.

Accumulating evidence suggests that soluble α-syn oligomers play an important role in the pathogenesis of genetic and idiopathic forms of PD [13], [15], [17]. PCAs provide the distinct advantage of allowing real time detection and analysis of oligomeric species of α-syn in live cells [14], [16], [19]. We have previously shown that CHIP reduces the amount of α-syn oligomers using a PCA which utilized α-syn tagged with GFP fragments [14]. GFP-based PCAs are irreversible which can be useful to study the formation of stabilized α-syn oligomers [34] but may be less helpful for the study of the kinetics of oligomer stability. Using a luciferase-based assay in which oligomerization of α-syn is transient [34], we found that CHIP mitigated the accumulation of soluble α-syn oligomers. In contrast, targeted knockdown of CHIP or blocking α-syn ubiquitinylation by mutating its lysine residues enhanced α-syn oligomerization. Thus, we infer that CHIP-mediated ubiquitinylation of α-syn may contribute, at least in part, to the reduction of α-syn oligomers. Furthermore we demonstrated that BAG5, which inhibits CHIP-mediated ubiquitinylation of α-syn in cells and *in vitro*, mitigates the effect of CHIP on reducing α-syn oligomers.

Other members of the BAG family of co-chaperone proteins, including BAG1 [23] and BAG2 [32], [33], have been previously found to interact with and regulate CHIP function. However, BAG1 and BAG2 have different effects on CHIP. BAG2 has been demonstrated to inhibit CHIP E3 ubiquitin ligase activity, resulting in decreased ubiquitinylation of CHIP substrates including Hsp70, the NBD1-R binding domain of cystic fibrosis transmembrane conductance regulator (CFTR), and raf-1 [32], [33]. BAG2 may inhibit CHIP function by disrupting the interaction between CHIP and its E2, UbcH5b [32], [33]. We found that, like BAG2, BAG5 inhibits the E3 ubiquitin ligase activity of CHIP. The mechanism by which BAG5 inhibits CHIP remains to be elucidated. By contrast, BAG1 has been shown to enhance CHIP-mediated degradation of the glucocorticoid receptor, a known substrate of CHIP [23]. In addition to a BAG domain, BAG1 contains a ubiquitin-like domain which may mediate an interaction with the 26S proteasome [54]. It is postulated that BAG1 could facilitate proteasomal degradation of CHIP substrates, such as the glucocorticoid receptor, by simultaneously binding to CHIP and the proteasome [23], [54]. Thus, the differential effect of BAG1 on CHIP function compared to BAG2 and BAG5 may be conferred by the ubiquitin-like domain of BAG1. Alternatively, there are inherent structural differences between the BAG domain of BAG1 and the BAG domains of BAG2 and BAG5 [42], [55]–[57] which may impart distinct effects on the regulation of associated E3 ligases such as CHIP.

BAG proteins have been found to regulate other E3 ubiquitin ligases in addition to CHIP. BAG1 has been previously shown to interact via its BAG domain with SIAH and inhibit SIAH function [58]. We have previously demonstrated that BAG5 directly interacts with and inhibits parkin [11], an E3 ubiquitin ligase which is mutated in an autosomal recessive form of PD. Thus, BAG domain-containing co-chaperones may be common regulators of E3 ubiquitin ligases. More recent evidence has shown that the ratio of the BAG domain-containing proteins BAG1 and BAG3 may shift protein degradation towards different pathways [59] and thus regulate proteostasis. We have previously shown that CHIP can target α-syn for degradation by both proteasomal and lysosomal pathways [10] and perhaps the ratios of different BAG domain-containing family members available for interacting with CHIP, such as BAG1, BAG2, and BAG5, may act as molecular switches in determining the ultimate fate of CHIP substrates such as α-syn.

In summary, we have identified α-syn as a novel substrate of CHIP E3 ubiquitin ligase activity and discovered that CHIP and α-syn form a protein complex with the co-chaperone BAG5. The functional significance of the CHIP-BAG5 interaction is the ubiquitinylation of α-syn by CHIP which may, in part, play a role in the reduction of α-syn oligomers. Consistent with this, we found that BAG5 inhibited CHIP-mediated ubiquitinylation of α-syn and mitigated the ability of CHIP to decrease levels of α-syn oligomers. Emerging evidence is suggesting that the BAG family of proteins, in general, may be critical regulators of E3 ubiquitin ligases. BAG domain-containing proteins in cooperation with other molecular chaperones, such as CHIP and Hsp70, may contribute to the triage of misfolded proteins but, in a disease state like PD, these critical protein handling pathways may become dysregulated. Thus, therapies which enhance CHIP E3 ubiquitin ligase activity or inhibit BAG5 function within dopaminergic neurons and other neuronal populations susceptible to α-syn-mediated toxicity may decrease cell death and slow the progression of neurodegeneration in PD.

## Materials and Methods

### DNA Constructs

The following DNA expression constructs were used: syn-luc1 and syn-luc2 [16], [19], linker-luc1 containing amino acids 1 to 93 of *Gaussia princeps* luciferase (kindly provided by S. Michnick, University of Montreal) [34], full-length native *Gaussia princeps* luciferase (pUC18 GLuc from Prolume), pcDNA3-CHIP, pcDNA3-CHIP ΔU, pcDNA3-CHIP ΔTPR (kind gifts from C. Patterson, University of North Carolina School of Medicine) [22], pcDNA3.1-myc-CHIP [10], pRK5-HA-Ubiquitin-WT and pRK5-HA-Ubiquitin-KO (Addgene) [60], pDEST-FLAG-BAG5 [11], pcDNA3.1-Hsp70 [8], and pcDNA3.1 (Invitrogen). To generate the synKR-luc1 and synKR-luc2 constructs, a coding sequence was designed in which all lysines in human full-length wild-type α-syn were substituted with arginine using codons to minimize sequence differences between wild-type and mutant. The sequence was synthesized and cloned into pDONR (DNA 2.0) and then subcloned into the same vectors used to make the syn-luc1 and syn-luc2 constructs.

### Cell Culture and Transfections

Human H4 neuroglioma cells (ATCC) were maintained in Opti-MEM media (Invitrogen) supplemented with 10% FBS (Invitrogen) at 37°C and 5% CO_2_. Cells were transiently transfected using SuperFect (Qiagen) according to the manufacturer's instructions. To generate cell lines stably expressing shRNA, H4 cells were transfected with MISSION shRNA pLKO.1-puro vectors encoding shRNAs targeting human CHIP (validated clones NM_005861.1-716s1c1 and NM_005861.1-479s1c1) or MISSION pLKO.1-puro vector containing a non-targeting control shRNA (Sigma). Cells were grown in selection media containing 4 µg/mL puromycin (Sigma) and colonies of cells demonstrating resistance to puromycin were isolated. The individual cell lines were maintained in Opti-MEM media supplemented with 10% FBS and 4 µg/mL puromycin. Protein expression in these cell lines was assessed by Western blot using anti-CHIP rabbit polyclonal antibodies (Calbiochem).

### Protein Complementation Assay with Gaussia Luciferase

H4 cells were transiently transfected in 6-well plates as described above. At 24 hr post-transfection, cells were scraped from each culture well in 1 mL PBS and then 100 µL of cells were transferred in triplicate to a 96-well plate. Native coelenterazine (Prolume), a cell permeable substrate of *Gaussia* luciferase, was resuspended in methanol to 1 mg/mL and dispensed per well to a final concentration of 20 µM by an automated plate reader, Wallac 1420 Victor2 (Perkin Elmer). The bioluminescent signal generated by the luciferase enzyme was integrated over 2 sec before measurement at 480 nm. Co-transfection of pSV-β-galactosidase vector (Promega) was used to normalize for transfection efficiency using the β-galactosidase enzyme assay system following the lysis of the remaining cells with reporter lysis buffer as per the manufacturer's protocol (Promega).

### Ubiquitinylation Assays

To test for ubiquitinylation in cells, H4 cells were transiently transfected with DNA expression constructs as indicated. At 24 hr post-transfection, cells were harvested under denaturing conditions in buffer containing 50 mM Tris (pH 7.4), 140 mM NaCl, 1% SDS and sheared with a 27-gauge needle 5 to 6 times. Samples were boiled for 5 min and then diluted 10-fold with buffer containing 50 mM Tris (pH 7.4), 140 mM NaCl, 1% Triton X-100, and protease inhibitor cocktail (Roche). Samples were centrifuged at 21,000× g for 2 min. Cell lysates were then incubated with anti-*Gaussia* luciferase rabbit polyclonal (Prolume) or anti-α-syn mouse monoclonal (BD Biosciences) antibodies overnight at 4°C. Immune complexes were isolated by the addition of 50 µL of protein A or G Sepharose beads (GE Healthcare) followed by incubation for 2 to 3 hr at 4°C. Immunoprecipitates were washed 3× with buffer containing 50 mM Tris (pH 7.4) and 500 mM NaCl, and then analyzed by SDS-PAGE and Western blot with anti-HA rat monoclonal (Roche) or anti-α-syn mouse monoclonal antibodies (Syn-1 from BD Biosciences or H3C, a kind gift from D. Clayton and J. George, University of Illinois) [61].

For *in vitro* ubiquitinylation assays, purified recombinant human α-syn (1.5 µM) from rPeptide was incubated with 1 µM His-tagged CHIP (Millipore) or HDM2 or MuRF1 (Boston Biochem), 0.5 µM Hsp70 (Assay Designs), 5 µM UbcH5b, 50 nM Ube1, 10 µM ubiquitin (Boston Biochem), and Mg-ATP (Boston Biochem) as indicated in 1× ubiquitin conjugation reaction buffer (Boston Biochem) for 1 hr at 30°C. GST or GST-BAG5 (0.5 to 4 µM) was included in the assays performed to test the effect of BAG5 on CHIP E3 activity. Prior to use in these assays, the GST fusion proteins were eluted from glutathione Sepharose beads and dialyzed in PBS using Slide-A-Lyzer dialysis cassettes (Pierce). Samples from the ubiquitinylation assays were analyzed by SDS-PAGE followed by Western blot with the following antibodies: anti-ubiquitin rabbit polyclonal (Dako), anti-α-syn mouse monoclonal (BD Biosciences), anti-CHIP rabbit polyclonal (Calbiochem), anti-Hsp70 rabbit polyclonal (Assay Designs), anti-His mouse monoclonal (GE Healthcare).

### Co-immunoprecipitations

Immunoprecipitation of proteins was performed under non-denaturing conditions from H4 cell lysates or mouse brain homogenates. H4 neuroglioma cells were lysed 24 hr after transfection in modified RIPA buffer (50 mM Tris (pH 7.4), 150 mM NaCl, 1 mM EDTA, 1% Igepal CA-360) with protease inhibitor cocktail (Roche). Cell lysates were incubated overnight at 4°C with agarose conjugated anti-FLAG M2 antibodies (Sigma) and washed according to the manufacturer's protocol. Samples were then separated by SDS-PAGE and analyzed by Western blot. Whole brains from C57BL/6J mice were homogenized in sucrose buffer (4 mM HEPES (pH 7.3), 0.32 M sucrose) supplemented with protease inhibitors (Roche) and then centrifuged at 1000× g for 15 min at 4°C followed by a subsequent centrifugation of the supernatant at 10,000× g for 15 min at 4°C. Homogenates were incubated overnight at 4°C with anti-α-syn antibodies (BD Biosciences) or control mouse IgG (Sigma). Immunoprecipitates were incubated with protein G Sepharose beads (GE Healthcare) for 4 hr at 4°C and then washed 3× with RIPA buffer supplemented with protease inhibitors. Samples were then separated by SDS-PAGE and analyzed by Western blot using HRP-conjugated IgG secondary antibodies (GE Healthcare) to detect α-syn, CHIP, and Hsp70, and biotin-conjugated IgG secondary antibodies (Jackson ImmunoResearch) to detect BAG5.

### GST Pull Down Assays

GST fusion constructs (GST, GST-BAG5, GST-BAG5 DARA [11]) were transformed into *E. coli* BL21 cells. GST fusion proteins were affinity purified using glutathione Sepharose beads (GE Healthcare) according to the manufacturer's instructions. Equal amounts of GST fusion proteins coupled to beads were incubated overnight at 4°C with proteins solubilized from transfected H4 cells lysed in modified RIPA buffer. Beads were then washed 3× with PBS. To test for direct binding between proteins, equal amounts of GST fusion proteins coupled to beads were incubated for 2 hr at 4°C with recombinant CHIP (100 ng) (Millipore) with or without Hsp70 (100 ng) (StressGen) in binding buffer (20 mM Tris (pH 8.0), 300 mM NaCl, 2 mM EDTA, 2 mM dithiothreitol). Samples were then washed 3× with binding buffer containing 400 mM NaCl. Samples were separated by SDS-PAGE and analyzed by Western blot.

### Immunohistochemistry

Human midbrain tissue from two subjects with a neuropathological diagnosis of DLB was obtained from the Harvard Brain Tissue Resource Center. The subjects were females aged 77 and 85 with a post-mortem interval of 5 and 12 hours, respectively. The tissue was fixed in 4% paraformaldehyde and sectioned as free floating 40 µm sections. The sections were permeabilized in 0.5% Triton X-100 in TBS (pH 7.4) for 10 min at room temperature. The sections were then blocked in 3% normal goat serum (Jackson ImmunoResearch) for 1 hr. Sections were incubated in blocking solution at 4°C overnight with a combination of mouse anti-α-syn antibody (dilution 1∶250; BD Bioscience) and rabbit anti-CHIP antibody (dilution 1∶200; Calbiochem), or with a combination of mouse anti-BAG5 antibody (dilution 1∶250; Santa Cruz) [11] and rabbit anti-CHIP antibody (dilution 1∶200; Calbiochem). On the following day, sections were washed three times in TBS, followed by incubation with secondary antibodies conjugated to AlexFluor488 (dilution 1∶500; Invitrogen) or Cy3 (dilution 1∶500; Jackson) for 1 hr at room temperature. Sections were subsequently washed with TBS and mounted on slides with Vectashield mounting media with DAPI (Vector Labs). Cells were imaged on an Olympus BX51 microscope with an epifluorescence attachment. To distinguish positive staining from autofluorescence or non-specific staining, sections were incubated with no antibodies or with non-immune sera (mouse IgG and rabbit IgG from Jackson ImmunoResearch) in place of primary antibodies, respectively. These control sections were examined with the same intensity setting and exposure times used for the labelled samples.
